# Supplementary material for: Measurement instruments for sexual identity minority stress in adults: A scoping review
Source: PLoS One. 2026 Feb 12;21(2):e0342420. doi: 10.1371/journal.pone.0342420 (PMC12900343; doi:10.1371/journal.pone.0342420)
Supplement: S2 File — Search Strategy. (DOCX) [file pone.0342420.s002.docx]

Supplement 2

The PRISMA flow diagram displays the combined results of four separate literature searches. The update searches were conducted independently. All search results were pooled, and duplicates were removed.

Ovid - MEDLINE(R)

Ovid - Medline ALL 1946 to August 24, 2021 on 25.08.2021 (without time restrictions)

First update:
Ovid - Medline ALL 1946 to October 05, 2022 on 06.10.2022 (from 24.08.2021 to 05.10.2022)

Second update:
Ovid - Medline ALL 1946 to January 30, 2024 on 31.01.2024 (from 05.10.2022 to 30.01.2024)

Third update:
Ovid - Medline ALL 1946 to February 11, 2025 on 11.02.2025 (from 30.01.2024 to 07.02.2025)

Filter: Measurement properties filter / Exclusion filter; Version 2. CB Terwee at al.,2009^[[1]](#footnote-1)^: *adjustments to changes in MeSH terms and translation to Ovid-Medline (no validated)*

Table S2. Search strategy - MEDLINE

| **#** | **Search string** |
| --- | --- |
| 1 | exp "Sexual and Gender Minorities"/ |
| 2 | ((gender or sexual or stress) adj3 minorit*).ti,ab,kw. |
| 3 | (GLBT or GLBTQ or LBG or LGBT or LGBTQ or LGBQ or TGNC or LGBTQI or LGBTQIA or LGBTI or LGBTIQ).ti |
| 4 | exp bisexuality/ or exp homosexuality/ or exp homosexuality, female/ or exp homosexuality, male/ or exp transgender persons/ or exp transsexualism/ |
| 5 | (bisexual* or lesbian* or gay* or Lesbigay or queer* or homosexual* or transgender* or transsexual* or pansexual* or asexual* or intersex* or agender or genderqueer or genderfluid or gender-fluid or gender-variant or nonbinar* or non-binar* or nonheteronormativ* or transident* or gender-nonconform* or nonheterosexual* or non-heterosexual*).ti,ab,kw. |
| 6 | (GLBT or GLBTQ or LBG or LGBT or LGBTQ or LGBQ or TGNC or LGBTQI or LGBTQIA or LGBTI or LGBTIQ).ti,ab,kw or (gender adj3 (identity or identification or diversity)).ti,ab,kw |
| 7 | exp "minority health"/ or exp "mental health"/ or exp "Health Status"/ or exp "social stigma"/ or exp "shame"/ or exp "social discrimination"/ |
| 8 | (((mental or status or level or social) adj3 (health or stress)) or (minorit* or stigma or stigmata or stigmatisation or stigmatiz* or gender bias or discriminat* or sexism or socio* or homophob* or prejudice)).ti,ab,kw. |
| 9 | or/4-6 |
| 10 | 7 or 8 |
| 11 | 9 and 10 |
| 12 | or/1-3,11 |
| 13 | (instrumentation or methods).fs. or (validation study or comparative study).pt. or exp psychometrics/ or psychometr*.ti,ab. or clin#metr*.tw. or exp outcome assessment, Health Care/ or outcome assessment.ti,ab. or outcome measure*.tw. or exp observer variation/ or exp Health Status Indicators/ or exp "Reproducibility of Results"/ or exp Discriminant Analysis/ or (observer variation or (reproducib* or (reliab* or (unreliab* or (valid* or (coefficient or (homogeneity or (homogeneous or (internal consistency or (cronbach* and alpha?)))))))))).ti,ab. or (item and (correlation* or selection* or reduction*)).ti,ab. or (agreement or (precision or (imprecision or precise values))).tw. or (test-retest or (test and retest) or (reliab* and (test or retest))).ti,ab. or (stability or int?r?rater or int?r?-rater or int?r?tester or int?r?-tester or int?r?observer or int?r?-observer or int?r?technician or int?r?-technician or int?r?examiner or int?r?-examiner or int?r?assay or int?r?-assay or int?r?individual or int?r?-individual or int?r?participant or int?r?-participant or kappa or kappas or kappa's).ti,ab. or (repeatab* or ((replicab* or repeated) and (measure? or finding? or result? or test? tests))).tw. or (generali#a* or concordance or (intraclass and correlation*) or discriminative or known group or factor analys#s or factor structure? or dimension* or subscale*).ti,ab. or ((multitrait and scaling and analys#s) or item discriminant or interscale correlation* or error? or individual variability or interval variability or rate variability).ti,ab. or ((variability and (analys#s or values)) or (uncertainty and (measurement or measuring))).ti,ab. or ("standard error of measurement" or sensitiv* or responsive* or (limit and detection) or "minimal detectable concentration" or interpretab*).ti,ab. or ((minimal or minimally or clinical or clinically) and (important or significant or detectable) and (change or difference)).ti,ab. or (small* and (real or detectable) and (change or difference)).ti,ab. or ("meaningful change" or "ceiling effect" or "floor effect" or "Item response model" or IRT or Rasch or "Differential item functioning" or DIF or "computer adaptive testing" or "item bank" or "cross-cultural equivalence").ti,ab. |
| 14 | 12 and 13 |
| 15 | (("delphi-technique" or cross-sectional).ti. or addresses.pt. or biography.pt. or "case reports".pt. or comment.pt. or directory.pt. or editorial.pt. or festschrift.pt. or interview.pt. or lecture.pt. or "legal case".pt. or legislation.pt. or letter.pt. or news.pt. or "newspaper article".pt. or "patient education handout".pt. or "popular work".pt. or congress.pt. or "consensus development conference".pt. or "consensus development conference, nih".pt. or "practice guideline".pt.) not (exp animals/ not exp humans/) |
| 16 | 14 not 15 |

**Embase (Ovid)**

**Embase (OVID) 1974 to 2021 August 25 on 25.08.2021** (without time restrictions)

**First update:
Embase (OVID) 1974 to 2022 October 07 on 09.10.2022** (from 24.08.2021 to 07.10.2022)

**Second update:
Embase (OVID) 1974 to 2024 January 30 on 31.01.2024** (from 07.10.2022 to 30.01.2024)

**Third update:
Embase (OVID) 1974 to 2025 February 11 on 11.02.2025** (from 30.01.2024-11.02.2025)

Filter: Measurement properties filter / Exclusion filter; Version 2. CB Terwee at al.,2009^1^:
*translation to Ovid-Embase (no validated)*

Table S3. Search strategy - Embase

| **#** | **Search string** |
| --- | --- |
| 1 | "Sexual and Gender Minorities"/ |
| 2 | ((gender or sexual or stress) adj3 minorit*).ti,ab,kw. |
| 3 | (GLBT or GLBTQ or LBG or LGBT or LGBTQ or LGBQ or TGNC or LGBTQI or LGBTQIA or LGBTI or LGBTIQ).ti. |
| 4 | exp lgbtqia+ people/ or exp bisexuality/ or exp homosexuality/ or exp transgender/ or exp transsexualism/ |
| 5 | (bisexual* or lesbian* or gay* or lesbigay or queer* or homosexual* or transgender* or transsexual* or pansexual* or asexual* or intersex* or agender or genderqueer or genderfluid or gender-fluid or gender-variant or nonbinar* or non-binar* or nonheteronormativ* or transident* or gender-nonconform* or nonheterosexual* or non-heterosexual*).ti,ab,kw. |
| 6 | (GLBT or GLBTQ or LBG or LGBT or LGBTQ or LGBQ or TGNC or LGBTQI or LGBTQIA or LGBTI or LGBTIQ or (gender adj3 (identity or identification or diversity))).ti,ab,kw. |
| 7 | exp "minority health"/ or exp "gender bias"/ or exp "mental health"/ or exp "health status"/ or exp "social stigma"/ or exp "shame"/ or exp "social discrimination"/ |
| 8 | (((mental or status or level or social) adj3 (health or stress)) or (minorit* or stigma or stigmata or stigmatisation or stigmatiz* or gender bias or discriminat* or sexism or socio* or homophob* or prejudice)).ti,ab,kw. |
| 9 | or/4-6 |
| 10 | 7 or 8 |
| 11 | 9 and 10 |
| 12 | or/1-3,11 |
| 13 | exp intermethod comparison/ or exp data collection method/ or exp validation study/ or exp comparative study/ or exp feasibility study/ or exp pilot study/ or exp psychometry/ or psychometr*.ti,ab. or clin#metr*.tw. or exp outcome assessment/ or outcome assessment.ti,ab. or outcome measure*.tw. or exp observer variation/ or exp Health Status Indicators/ or exp reproducibility/ or exp Discriminant Analysis/ or (observer variation or (reproducib* or (reliab* or (unreliab* or (valid* or (coefficient or (homogeneity or (homogeneous or (internal consistency or (cronbach* and alpha?)))))))))).ti,ab. or (item and (correlation* or selection* or reduction*)).ti,ab. or (agreement or (precision or (imprecision or precise values))).tw. or (test-retest or (test and retest) or (reliab* and (test or retest))).ti,ab. or (stability or int?r?rater or int?r?-rater or int?r?tester or int?r?-tester or int?r?observer or int?r?-observer or int?r?technician or int?r?-technician or int?r?examiner or int?r?-examiner or int?r?assay or int?r?-assay or int?r?individual or int?r?-individual or int?r?participant or int?r?-participant or kappa or kappas or kappa's).ti,ab. or (repeatab* or ((replicab* or repeated) and (measure? or finding? or result? or test? tests))).tw. or (generali#a* or concordance or (intraclass and correlation*) or discriminative or known group or factor analys#s or factor structure? or dimension* or subscale*).ti,ab. or ((multitrait and scaling and analys#s) or item discriminant or interscale correlation* or error? or individual variability or interval variability or rate variability).ti,ab. or ((variability and (analys#s or values)) or (uncertainty and (measurement or measuring))).ti,ab. or ("standard error of measurement" or sensitiv* or responsive* or (limit and detection) or "minimal detectable concentration" or interpretab*).ti,ab. or ((minimal or minimally or clinical or clinically) and (important or significant or detectable) and (change or difference)).ti,ab. or (small* and (real or detectable) and (change or difference)).ti,ab. or ("meaningful change" or "ceiling effect" or "floor effect" or "Item response model" or IRT or Rasch or "Differential item functioning" or DIF or "computer adaptive testing" or "item bank" or "cross-cultural equivalence").ti,ab. |
| 14 | 12 and 13 |
| 15 | (("delphi-technique" or cross-sectional or "case report?" or comment).ti. or editorial.pt. or letter.pt. or consensus development/ or practice guideline/ or conference abstract/ or symposium/ or workshop/ or conference abstract.pt. or congress??.ti.) not (exp animals/ not exp humans/) |
| 16 | 14 not 15 |
| 17 | limit 16 to embase |
| 18 | limit 16 to medline |
| 19 | 17 or 18 |
| 20 | 16 not 19 |
| 21 | 17 or 20 |

PsycInfo (OVID)

APA PsycInfo 1806 to August Week 3 2021; on 24.08.2021 (without time restrictions)

First update:
APA PsycINFO 1806 to October Week 1 2022; on 09.10.2022 (publ. year: 2021, 2022)

Second update:
APA PsycINFO 1806 to January Week 4 2024; on 31.01.2024 (publ. year: 2022, 2023, 2024)

Third update:
APA PsycINFO 1806 to February 2025 Week 1; am 11.02.2025 (publ. year: 2024, 2025)

Filter: Measurement properties filter / Exclusion filter; Version 2. CB Terwee at al.,2009^1^:

*translation to Ovid-PsycInfo (no validated)*

Table S4. Search strategy - PsycInfo

| **#** | **Search string** |
| --- | --- |
| 1 | exp Minority Stress/ |
| 2 | exp Sociosexual Orientation/ |
| 3 | ((gender or sexual or stress) adj3 minorit*).ti,ab. |
| 4 | (GLBT or GLBTQ or LBG or LGBT or LGBTQ or LGBQ or Lesbigay or TGNC or LGBTQI or LGBTQIA or LGBTI or LGBTIQ).ti. |
| 5 | exp gender identity/ or exp sexual minority groups/ or Sexual Orientation/ or Asexuality/ |
| 6 | (bisexual* or lesbian* or gay* or lesbigay or queer* or homosexual* or transgender* or transsexual* or pansexual* or asexual* or intersex* or agender or genderqueer or genderfluid or gender-fluid or gender-variant or nonbinar* or non-binar* or nonheteronormativ* or transident* or gender-nonconform* or nonheterosexual* or non-heterosexual*).ti,ab. |
| 7 | (GLBT or GLBTQ or LBG or LGBT or LGBTQ or LGBQ or TGNC or LGBTQI or LGBTQIA or LGBTI or LGBTIQ or (gender adj3 (identity or identification or diversity))).ti,ab. |
| 8 | exp stigma/ or social discrimination/ or exp Sexual Attitudes/ or exp emotional states/ or mental status/ |
| 9 | (((mental or status or level or social) adj3 (health or stress)) or (minorit* or stigma or stigmata or stigmatisation or stigmatiz* or gender bias or discriminat* or sexism or socio* or homophob* or prejudice)).ti,ab. |
| 10 | or/5-7 |
| 11 | 8 or 9 |
| 12 | 10 and 11 |
| 13 | or/1-4,12 |
| 14 | ("Psychometrics & Statistics & ‎Methodology" or "Research ‎Methods & Experimental Design").cw. or "Measurement".sh. or "Error ‎Analysis".sh. or "Test ‎Construction".sh. or "Interrater ‎Reliability".sh. or "Content ‎Analysis".sh. or "Error of ‎Measurement".sh. or "Factor ‎Structure".sh. or "Testing ‎Methods".sh. or "Statistical ‎Reliability".sh. or "Consistency (Measurement)".sh. or "Computer ‎Assisted Testing".sh. or "Factor ‎Analysis".sh. or "Prediction".sh. or "Statistical ‎Validity".sh. or "Prediction ‎Errors".sh. or (psychometr* or psychometr* or clinimetr* or clinometr* or "outcome ‎assessment" or "outcome ‎measure*" or "observer variation" or reproducib* or reliab* or unreliab* or valid* or coefficient or homogeneity or homogeneous or "internal consistency" or agreement or precision or imprecision or "precise ‎values" or (test-retest or reliab* or stability or int?r?rater or int?r?-rater or int?r?tester or int?r?-tester or int?r?observer or int?r?-observer or int?r?technician or int?r?-technician or int?r?examiner or int?r?-examiner or int?r?assay or int?r?-assay or int?r?individual or int?r?-individual or int?r?participant or int?r?-participant) or (kappa or kappas or kappa's or repeatab* or generali#a* or concordance) or (discriminative or known group or factor analys#s or dimension* or subscale*) or ("item discriminant" or "interscale correlation*" or error? or "individual variability") or ("standard error of measurement" or sensitiv* or responsive* or "meaningful change" or "ceiling effect" or "floor effect" or "Item response model" or IRT or Rasch or "Differential item functioning" or DIF or "computer adaptive testing" or "item bank" or "cross-cultural equivalence") or ("cronbach* alpha?" or "replicab* measure*" or "replicab* finding*" or "replicab* ‎result*" or "‎‎replicab* ‎test*") or ("repeated measure*" or "repeated finding*" or "repeated ‎result*" or "repeated test*" or "item correlation*" or "item ‎selection*" or "item reduction*") or ("Test retest" or "intraclass ‎correlation*" or "multitrait scaling ‎analys*" or "uncertainty measur*" or "variability ‎analys#s" or "variability value*" or "minimal* ‎important change") or ("minimal* ‎important difference" or "minimal* ‎significant change" or "minimal* ‎significant difference" or "minimal* ‎detectable ‎change" or "minimal* ‎detectable difference" or "clinical* ‎important change" or "clinical* ‎important ‎difference" or "clinical* ‎significant change" or "clinical* ‎significant difference" or "clinical* ‎detectable ‎change" or "clinical* ‎detectable difference" or "small* ‎real change" or "small* real ‎difference" or "small* detectable ‎change" or "small* detectable ‎difference")).ti,ab. |
| 15 | 13 and 14 |

PSYNDEX (OVID)

PSYNDEXplus Tests 1945 to May 2021; on 24.08.2021 (without time restrictions)

**First update:
PSYNDEXplus Tests 1945 to May 2022; on 09.10.2022** (without time restrictions)

**Second update:
PSYNDEXplus Tests 1945 to November 2023; on 31.01.2024** (without time restrictions)

**Third update:
PSYNDEXplus Tests 1945 to November 2024; on 11.02.2025** (without time restrictions)

Table S5. Search strategy - PSYNDEX

| **#** | **Search string** |
| --- | --- |
| 1 | ((gender or sexual or health or stress) and minorit*).ab,cw,kx,hw,ti. |
| 2 | exp gender identity/ or exp sexual minority groups/ or Sexual Orientation/ |
| 3 | (GLBT or GLBTQ or LBG or LGBT or LGBTQ or LGBQ or TGNC or LGBTQI or LGBTQIA or LGBTI or LGBTIQ or (gender adj3 (identity or identification or diversity))).ab,cw,kx,hw,ti. |
| 4 | exp Stigma/ |
| 5 | exp Social Discrimination/ |
| 6 | (stigma* or discriminat* or diskriminier*).ab,kx,ti. |
| 7 | (bisexu* or lesbi* or gay* or lesbigay or queer* or homosexu* or transgender* or transsexu* or pansexu* or asexu* or intersex* or agender or genderqueer or genderfluid or gender-fluid or gender-variant or nonbin* or non-bin* or nonheteronormativ* or transident* or gender-nonconform* or nonheterosexu* or non-heterosexu*).ab,cw,kx,hw,ti. |
| 8 | minderheit*.ab,cw,kx,hw,ti. |
| 9 | 1 or 2 or 3 or 4 or 5 or 6 or 7 or 8 |

Pascal and Francis (1972 to 2015); on 04.11.2021 (<https://pascal-francis.inist.fr/home/>)

*Advanced Search*

Table S6. Search strategy - Pascal and Francis

| **#** | **Search string** |
| --- | --- |
| 1 | ("Sexual and Gender Minority") OR ("Sexual and Gender Minorities") |
| 2 | ti.\*:(((gender OR sexual OR stress) AND minorit*)) OR kw.\*:(((gender OR sexual OR stress) AND minorit*)) |
| 3 | ti.\*:(GLBT OR GLBTQ OR LBG OR LGBT OR LGBTQ OR LGBQ OR TGNC OR LGBTQI OR LGBTQIA OR LGBTI OR LGBTIQ) OR kw.\*:(GLBT OR GLBTQ OR LBG OR LGBT OR LGBTQ OR LGBQ OR TGNC OR LGBTQI OR LGBTQIA OR LGBTI OR LGBTIQ) |
| 4 | (ti.\*:(gender) AND ti.\*:(identity or identification or diversity)) OR (kw.\*:(gender) AND kw.\*:(identity or identification or diversity)) |
| 5 | ti.\*:(bisexual* OR lesbian* OR gay* OR Lesbigay OR queer* OR homosexual* OR transgender* OR transsexual* OR pansexual* OR asexual* OR intersex* OR agender OR genderqueer OR genderfluid OR (gender AND fluid) OR (gender AND variant) OR nonbinar* OR (non AND binar*) OR nonheteronormativ* OR transident* OR (gender AND nonconform*) OR nonheterosexual* OR (non AND heterosexual*)) OR kw.\*:(bisexual* OR lesbian* OR gay* OR Lesbigay OR queer* OR homosexual* OR transgender* OR transsexual* OR pansexual* OR asexual* OR intersex* OR agender OR genderqueer OR genderfluid OR (gender AND fluid) OR (gender AND variant) OR nonbinar* OR (non AND binar*) OR nonheteronormativ* OR transident* OR (gender AND nonconform*) OR nonheterosexual* OR (non AND heterosexual*)) |
| 6 | (ti.\*:(minorit* OR stigma OR stigmata OR stigmatisation OR stigmatiz* OR "gender bias" OR discriminat* OR sexism OR socio* OR homophob* OR prejudice) OR kw.\*:(minorit* OR stigma OR stigmata OR stigmatisation OR stigmatiz* OR "gender bias" OR discriminat* OR sexism OR socio* OR homophob* OR prejudice)) |
| 7 | (#4 OR #5) AND #6 |
| 8 | (cc.\*:("002A01")) OR (cc.\*:("002A26A")) OR (cc.\*:("002A26B")) OR (cc.\*:("002A26C")) OR (cc.\*:("002A26G")) OR (cc.\*:("002A26M")) OR (cc.\*:("002B18A")) OR (cc.\*:("002B18B")) OR (cc.\*:("002B18H")) OR (cc.\*:("002B31")) OR (cc.\*:("521-10")) OR (cc.\*:("521-12")) OR (cc.\*:("521-13")) OR (cc.\*:("521-14")) OR (cc.\*:("521-25")) OR (cc.\*:("521-27A")) OR (cc.\*:("521-29A")) OR (cc.\*:("521-33")) OR (cc.\*:("521-41")) OR (cc.\*:("521-63")) OR (cc.\*:("770-B01")) OR (cc.\*:("770-B02")) OR (cc.\*:("770-B03")) OR (cc.\*:("770-B13")) OR (cc.\*:("770-D01")) OR (cc.\*:("770-D02")) OR (cc.\*:("770-D08B")) OR (cc.\*:("770-D08C")) |
| 9 | #7 AND #8 |
| 10 | 1 OR #2 OR #3 OR #9 |

**PTSDpub (Pilot) / ProQuest** ([https://www.ptsd.va.gov/ptsdpubs/searc h_ptsdpubs.asp](https://www.ptsd.va.gov/ptsdpubs/searc%20h_ptsdpubs.asp))

**PTSDpub / ProQuest 1871 to current; am 04.11.2021** (without time restrictions)

**First update:
PTSDpub / ProQuest 1871 to current; am 09.10.2022** (11.2021 to current)

**Second update:
PTSDpub / ProQuest 1871 to current; am 31.01.2024** (10.2022 to current)

**Third update:
PTSDpub / ProQuest 1871 to current; am 12.02.2025** (01.2024 to current)

*Advanced Search/ individual search steps*

Table S7. Search strategy - PTSDpub

| **#** | **Search string** | **Database limit** |
| --- | --- | --- |
| 1 | su("sexual and gender minority") |  |
| 2 | TI,IF((gender OR sexual OR stress) AND minorit*) | (durch Fachleute geprüfte Information=wissenschaftliche Zeitschriften) |
| 3 | TI,IF(GLBT OR GLBTQ OR LBG OR LGBT OR LGBTQ OR LGBQ OR TGNC OR LGBTQI OR LGBTQIA OR LGBTI OR LGBTIQ) | (durch Fachleute geprüfte Information=wissenschaftliche Zeitschriften) |
| 4 | TI,IF((gender AND (identity OR identification OR diversity)) OR bisexual* OR lesbian* OR gay* OR Lesbigay OR queer* OR homosexual* OR transgender* OR transsexual* OR pansexual* OR asexual* OR intersex* OR agender OR genderqueer OR genderfluid OR gender-fluid OR gender-variant OR nonbinar* OR non-binar* OR nonheteronormativ* OR transident* OR gender-nonconform* OR nonheterosexual* OR non-heterosexual*) AND TI,AB,IF(minorit* OR stigma OR stigmata OR stigmatisation OR stigmatiz* OR "gender bias" OR discriminat* OR sexism OR socio* OR homophob* OR prejudice) | (durch Fachleute geprüfte Information=wissenschaftliche Zeitschriften) |

**PubPsych** (<https://www.pubpsych.de/>)

**PubPsych on 04.11.2021** (without time restrictions)

**PubPsych on 09.10.2022** (PY = 2021, 2022)

**PubPsych on 31.01.2024** (PY = 2022, 2023, 2024)

**PubPsych on 12.02.2025** (PY = 2023, 2024, 2025)

*Advanced Search/ individual search steps / searchfield:* „Finde Publikation(en) mit allen Wörtern“ / *Database-limit - NOT DB="MEDLINE" NOT DB="Pascal" NOT DB="Psyndex"*

Table S8. Search strategy - PubPsych

| **Search string** |
| --- |
| ("Sexual and Gender Minority") NOT DB="MEDLINE" NOT DB="Pascal" NOT DB="Psyndex" |
| ("Sexual and Gender Minorities") NOT DB="MEDLINE" NOT DB="Pascal" NOT DB="Psyndex" |
| TI=(gender OR sexual OR stress) AND TI=(minorit*) NOT DB="MEDLINE" NOT DB="Pascal" NOT DB="Psyndex" |
| SW=(gender OR sexual OR stress) AND SW=(minorit*) NOT DB="MEDLINE" NOT DB="Pascal" NOT DB="Psyndex" |
| TI=(GLBT OR GLBTQ OR LBG OR LGBT OR LGBTQ OR LGBQ OR TGNC OR LGBTQI OR LGBTQIA OR LGBTI OR LGBTIQ) NOT DB="MEDLINE" NOT DB="Pascal" NOT DB="Psyndex" |
| AB=(GLBT OR GLBTQ OR LBG OR LGBT OR LGBTQ OR LGBQ OR TGNC OR LGBTQI OR LGBTQIA OR LGBTI OR LGBTIQ) NOT DB="MEDLINE" NOT DB="Pascal" NOT DB="Psyndex" |
| SW=(GLBT OR GLBTQ OR LBG OR LGBT OR LGBTQ OR LGBQ OR TGNC OR LGBTQI OR LGBTQIA OR LGBTI OR LGBTIQ) NOT DB="MEDLINE" NOT DB="MEDLINE" NOT DB="Pascal" NOT DB="Psyndex" |
| TI=(bisexual* OR lesbian* OR gay* OR Lesbigay OR queer* OR homosexual* OR transgender* OR transsexual* OR pansexual* OR asexual* OR intersex* OR agender OR genderqueer OR genderfluid OR gender-fluid OR gender-variant OR nonbinar* OR non-binar* OR nonheteronormativ* OR transident* OR gender-nonconform* OR nonheterosexual* OR non-heterosexual*) AND TI=(minorit* OR stigma OR stigmata OR stigmatisation OR stigmatiz* OR „gender bias“ OR discriminat* OR sexism OR socio* OR homophob* OR prejudice) NOT DB="MEDLINE" NOT DB="Pascal" NOT DB="Psyndex" |
| TI=(bisexual* OR lesbian* OR gay* OR Lesbigay OR queer* OR homosexual* OR transgender* OR transsexual* OR pansexual* OR asexual* OR intersex* OR agender OR genderqueer OR genderfluid OR gender-fluid OR gender-variant OR nonbinar* OR non-binar* OR nonheteronormativ* OR transident* OR gender-nonconform* OR nonheterosexual* OR non-heterosexual*) AND AB=(minorit* OR stigma OR stigmata OR stigmatisation OR stigmatiz* OR „gender bias“ OR discriminat* OR sexism OR socio* OR homophob* OR prejudice) NOT DB="MEDLINE" NOT DB="Pascal" NOT DB="Psyndex" |
| TI=((gender AND identity) OR (gender AND identification) OR (gender AND diversity)) AND TI=(minorit* OR stigma OR stigmata OR stigmatisation OR stigmatiz* OR „gender bias“ OR discriminat* OR sexism OR socio* OR homophob* OR prejudice) NOT DB="MEDLINE" NOT DB="Pascal" NOT DB="Psyndex" |
| TI=((gender AND identity) OR (gender AND identification) OR (gender AND diversity)) AND AB=(minorit* OR stigma OR stigmata OR stigmatisation OR stigmatiz* OR „gender bias“ OR discriminat* OR sexism OR socio* OR homophob* OR prejudice) NOT DB="MEDLINE" NOT DB="Pascal" NOT DB="Psyndex"– |
| TI=((gender AND identity) OR (gender AND identification) OR (gender AND diversity)) AND SW=(minorit* OR stigma OR stigmata OR stigmatisation OR stigmatiz* OR „gender bias“ OR discriminat* OR sexism OR socio* OR homophob* OR prejudice) NOT DB="MEDLINE" NOT DB="Pascal" NOT DB="Psyndex" |

**Web of Science**

**Web of Science: Core Collection (WOS.SSCI: 1956 to 2021, WOS.AHCI: 1975 to 2021, WOS.BHCI: 2013 to 2021, WOS.ESCI: 2017 to 2021, WOS.SCI: 1900 to 2021); on 10.12.2021** (without time restrictions)

**First update:
Web of Science: Core Collection (WOS.SSCI: 1956 to 2022, WOS.AHCI: 1975 to 2022, WOS.BHCI: 2013 to 2022, WOS.ESCI: 2017 to 2022, WOS.SCI: 1900 to 2022); on 07.10.2022;**(from 10.12.2021 to 07.10.2022)

**Second update:
Web of Science am 02.02.2024; Suche in Core Collection (WOS.SSCI: 1956 to 2024, WOS.AHCI: 1975 to 2024, WOS.BHCI: 2013 to 2024, WOS.ESCI: 2019 to 2024, WOS.SCI: 1900 to 2024)**(from 07.10.2022 to 02.02.2024)

**Third update:
Web of Science am 27.02.2025; Suche in Core Collection (WOS.SSCI: 1956 to 2025, WOS.AHCI: 1975 to 2025, WOS.BHCI: 2013 to 2025, WOS.ESCI: 2020 to 2025, WOS.SCI: 1900 to 2025)**(from 02.02.2024 to 27.02.2025)

*Advance Search*

Filter: Measurement properties filter / Exclusion filter; Version 2. CB Terwee at al.,2009^1^:
*Adjustments to changes in MeSH terms an translation to Web of sience with Polyglot Search* *SR-Accelerator (*[*https://sr-accelerator.com/#/polyglot*](https://sr-accelerator.com/#/polyglot)*)*

Table S9. Search strategy - Web of Science

| **#** | **Search string** |
| --- | --- |
| 1 | TS=("Sexual and Gender Minorities") |
| 2 | TI=((gender OR sexual OR stress) NEAR/3 minorit*) |
| 3 | TI=(GLBT OR GLBTQ OR LBG OR LGBT OR LGBTQ OR LGBQ OR TGNC OR LGBTQI OR LGBTQIA OR LGBTI OR LGBTIQ) |
| 4 | TI=(bisexual* OR lesbian* OR gay* OR Lesbigay OR queer* OR homosexual* OR transgender* OR transsexual* OR pansexual* OR asexual* OR intersex* OR agender OR genderqueer OR genderfluid OR gender-fluid OR gender-variant OR nonbinar* OR non-binar* OR nonheteronormativ* OR transident* OR gender-nonconform* OR nonheterosexual* OR non-heterosexual* ) OR AB=(bisexual* OR lesbian* OR gay* OR Lesbigay OR queer* OR homosexual* OR transgender* OR transsexual* OR pansexual* OR asexual* OR intersex* OR agender OR genderqueer OR genderfluid OR gender-fluid OR gender-variant OR nonbinar* OR non-binar* OR nonheteronormativ* OR transident* OR gender-nonconform* OR nonheterosexual* OR non-heterosexual*) |
| 5 | (TI=(GLBT OR GLBTQ OR LBG OR LGBT OR LGBTQ OR LGBQ OR TGNC OR LGBTQI OR LGBTQIA OR LGBTI OR LGBTIQ) OR AB=(GLBT OR GLBTQ OR LBG OR LGBT OR LGBTQ OR LGBQ OR TGNC OR LGBTQI OR LGBTQIA OR LGBTI OR LGBTIQ)) OR (TI=(gender NEAR/3 (identity OR identification OR diversity)) OR AB=(gender NEAR/3 (identity OR identification OR diversity))) |
| 6 | (TI=(((mental OR status OR level OR social) NEAR/3 (health OR stress)) OR (minorit* OR stigma OR stigmata OR stigmatization OR stigmatiz* OR "gender bias" OR discriminat* OR sexism OR socio* OR homophob* OR prejudice)) OR AB=(((mental OR status OR level OR social) NEAR/3 (health OR stress)) OR (minorit* OR stigma OR stigmata OR stigmatization OR stigmatiz* OR "gender bias" OR discriminat* OR sexism OR socio* OR homophob* OR prejudice))) |
| 7 | #1 OR #2 OR #3 |
| 8 | (#4 OR #5) AND #6 |
| 9 | #7 OR #8 |
| 10 | TS=("Instrumentation" OR "Methods" OR "Validation Stud*" OR "Comparative Stud*" OR psychometrics OR psychometr* OR clinimetr* OR clinometr* OR "outcome assessment" OR "outcome measure*" OR "observer variation" OR "observer variation" OR "Health Status Indicators" OR "reproducibility of results" OR reproducib* OR "discriminant analysis" OR reliab* OR unreliab* OR valid* OR "coefficient of variation" OR coefficient OR homogeneity OR homogeneous OR "internal consistency" OR (cronbach* AND (alpha OR alphas)) OR (item AND (correlation* OR selection* OR reduction*)) OR agreement OR precision OR imprecision OR "precise values" OR test-retest OR (test AND retest) OR (reliab* AND (test OR retest)) OR stability OR interrater OR inter-rater OR intrarater OR intra-rater OR intertester OR inter-tester OR intratester OR intra-tester OR interobserver OR inter-observer OR intraobserver OR intra-observer OR intertechnician OR inter-technician OR intratechnician OR intra-technician OR interexaminer OR inter-examiner OR intraexaminer OR intra-examiner OR interassay OR inter-assay OR intraassay OR intra-assay OR interindividual OR inter-individual OR intraindividual OR intra-individual OR interparticipant OR inter-participant OR intraparticipant OR intra-participant OR kappa OR kappa’s OR kappas OR repeatab* OR ((replicab* OR repeated) AND (measure OR measures OR findings OR result OR results OR test OR tests)) OR generaliza* OR generalisa* OR concordance OR (intraclass AND correlation*) OR discriminative OR "known group" OR "factor analysis" OR "factor analyses" OR "factor structure" OR "factor structures" OR dimension* OR subscale* OR (multitrait AND scaling AND (analysis OR analyses)) OR "item discriminant" OR "interscale correlation*" OR error OR errors OR "individual variability" OR "interval variability" OR "rate variability" OR (variability AND (analysis OR values)) OR (uncertainty AND (measurement OR measuring)) OR "standard error of measurement" OR sensitiv* OR responsive* OR (limit AND detection) OR "minimal detectable concentration" OR interpretab* OR ((minimal OR minimally OR clinical OR clinically) AND (important OR significant OR detectable) AND (change OR difference)) OR (small* AND (real OR detectable) AND (change OR difference)) OR "meaningful change" OR "ceiling effect" OR "floor effect" OR "Item response model" OR IRT OR Rasch OR "Differential item functioning" OR "DIF" OR "computer adaptive testing" OR "item bank" OR "cross-cultural equivalence") |
| 11 | #9 AND #10 |
| 12 | (ALL=addresses OR ALL=biography OR ALL="case reports" OR ALL=comment OR ALL=directory OR ALL=editorial OR ALL=festschrift OR ALL=interview OR ALL=lectures OR ALL="legal cases" OR ALL=legislation OR ALL=letter OR ALL=news OR ALL="newspaper article" OR ALL="patient education handout" OR ALL="popular works" OR ALL=congresses OR ALL="consensus development conference" OR ALL="consensus development conference, nih" OR ALL="practice guideline") NOT (ALL=animals NOT ALL=humans) |
| 13 | #11 NOT #12 |

GESIS Open Access Repository for Measurement Instruments (<https://zis.gesis.org/erweiterteSuche/>)**; on 24.11.2021; 09.10.2022; 31.01.2024**

*Advanced Search “all Searchfields”*

Table S10. Search strategy - GESIS Open Access Repository

| **Search string** |
| --- |
| Sexual und Minorit* |
| gender und minorit* |
| minorit* |
| (Sexuell* und Minderheit* |
| geschlecht* und minderheit* |
| Minderheitenstress |
| minderheit*= |
| GLBT* ODER LBG ODER LGBT* ODER LGBQ ODER TGNC |
| bisex* ODER lesbi* ODER gay* ODER schwul* ODER homosex* |
| queer* ODER gender* |
| transgender* ODER agender* |
| transsex* ODER pansex* ODER asex* ODER intersex* ODER nonbinar* ODER non-binar* ODER nonheteronormativ* ODER transident* ODER *heterosex* |
| stigma* ODER discriminat* ODER sexis* ODER homophob* ODER prejudice |
| diskrimin* ODER vorurteil*= |
| geschlechterrolle* ODER geschlechteridentit* ODER geschlechterorient*= |
| geschlecht* UND Identit* |

**Iterative hand search for grey literature am 07.12.2021** in der GLBT Alliance in Social and Personality Psycology (GASP) Measures Database, University of Utah, <https://gasp.psych.utah.edu/measures.php>

1. Terwee CB, Jansma EP, Riphagen II, de Vet HC. Development of a methodological PubMed search filter for finding studies on measurement properties of measurement instruments. Qual Life Res. 2009 Oct;18(8):1115-23. doi: 10.1007/s11136-009-9528-5. [↑](#footnote-ref-1)
