# Supplementary material for: Measurement instruments for sexual identity minority stress in adults: A scoping review
Source: PLoS One. 2026 Feb 12;21(2):e0342420. doi: 10.1371/journal.pone.0342420 (PMC12900343; doi:10.1371/journal.pone.0342420)
Supplement: S3 File — Data extraction form. (DOCX) [file pone.0342420.s003.docx]

Supplement 3

The following template was used to extract data presented in Table 1:

**Table S11. Data extraction template for Table 1**

| **No.** | \| **Questionnaire (Abbreviation)** \| \| --- \| \|  \| | **Language** | **Construct**  **(Subscale)** | **Validation Population** | **No. of Items (Subscale)** | **Study Context** | **Reference** |
| --- | --- | --- | --- | --- | --- | --- | --- | --- | --- |
|  |  |  |  |  |  |  |  |

The following templates were used for data analysis:

**Table S12. Data extraction template for analysis on measurement constructs**

| **Questionnaire (Abbreviation)** | **Internalized Homonegativity** | **Stigmatization** | **Other: Microaggressions** | **Other** | **Discrimination** | **Minority Stress** | **Identity Concealment** | **Community Connectedness** | **Self-Acceptance and Pride** |
| --- | --- | --- | --- | --- | --- | --- | --- | --- | --- |
|  | [0 or 1] | [0 or 1] | [0 or 1] | [0 or 1] | [0 or 1] | [0 or 1] | [0 or 1] | [0 or 1] | [0 or 1] |

**Table S13. Data extraction template for analysis on measurement populations**

| **Questionnaire (Abbreviation)** | **Sexual Identity** | | | **Gender** | | |
| --- | --- | --- | --- | --- | --- | --- |
|  | **Lesbian** | **Gay** | **Bisexual** | **Female** | **Male** | **Including Trans+** |
|  | [0 or 1] | [0 or 1] | [0 or 1] | [0 or 1] | [0 or 1] | [0 or 1] |
