# Supplementary material for: Measurement instruments for sexual identity minority stress in adults: A scoping review
Source: PLoS One. 2026 Feb 12;21(2):e0342420. doi: 10.1371/journal.pone.0342420 (PMC12900343; doi:10.1371/journal.pone.0342420)
Supplement: S4 File — Adaptions of measurement instruments. (DOCX) [file pone.0342420.s004.docx]

**Supplement 4**

**Table S14. Adaptions of measurement instruments**

| Adaption | \| Questionnaire (Abbreviation) \| \| --- \| | Language | Construct (Subscale) | Validation Population | No. of Items (Subscale) | Psychometric Study |
| --- | --- | --- | --- | --- | --- | --- | --- |
| Original | Lesbian and Gay Identity Scale (LGIS) | English | Need for Privacy, Need for Acceptance, Internalized Homonegativity, Difficult Process, Identity Confusion, Superiority | Lesbian or Gay | 6 + 5 + 5 + 5 + 4 + 2 | (1) |
| Adaption | Lesbian, Gay, and Bisexual Identity Scale (LGBIS) | English | Concealment and Negative Identity (Acceptance Concerns, Concealment Motivation, Identity Uncertainty, Internalized Homonegativity, Difficult Process, Identity Superiority, Identity Affirmation, Identity Centrality) | LGBQT | 27 (3 + 3 + 4 + 3 + 3 + 3 + 3 + 5) | (2) |
| Original | Internalized Homophobia Scale (IH Scale) | English | Internalized Homophobia (Public Identification as Gay, Perception of Stigma Associated with Being Gay, Social Comfort with Gay Men, Moral and Religious Acceptability of Being Gay) | MSM | 26 (10 + 6 + 6 + 4) | (3) |
| Adaption | Reactions to Homosexuality Scale (RHS) | English, Spanish | Internalized Homonegativity (Personal Comfort with a Gay Identity, Social Comfort with Gay Men, Public Identification as Gay) | MSM | 7 (3 + 2 + 2) | (4) |
| Original | Internalized Homophobia Scale (IHP) | English | Internalized Homophobia | Gay men | 9 | - ^[[1]](#footnote-1)^ |
| Adaption | Revised Internalized Homophobia (IHP-R) Scale | English | Internalized Sexual Stigma | LGB | 5 | (5) |
| Adaption | Internalized Homophobia Scale (IHS) | English | Internalized Homophobia | LGB | 9 | (6) |
| Adaption | Internalized Bi/Homophobia Scale (IBHS) | English | Internalized Bi-/Homophobia | Bisexual MSMW | 13 | (7) |
| Original | Nungesser Homosexual Attitudes Inventory (NHAI) | English | Internalized homophobia (Personal Homonegativity, Global Homonegativity, Disclosure) | Gay | 34 (10 + 12 + 12) | (8) |
| Adaption | Shidlo-revised Nungesser Homosexual Attitudes Inventory (NHAI-SR) | English | Internalized homophobia (Personal Homonegativity, Global Homonegativity, Disclosure) | Gay | 36 (14 + 9 + 13) | (9) |
| Original | Scale by Diaz et al. | English | Experiences of Homophobia | None | 11 | - ^[[2]](#footnote-2)^ |
| Adaption | The China MSM Stigma Scale | Chinese | Homosexuality Stigma (Perceived Stigma, Enacted Stigma) | MSM | 9 (3 + 6) | (10) |
| Adaption | The Neilands sexual stigma scale | English, Kiswahili | Homosexuality Stigma (Perceived Stigma, Enacted Stigma) | Gay and Bisexual MSM | 8 (3 + 5) | (11) |

**References**

1. Mohr JJ, Fassinger R. Measuring Dimensions of Lesbian and Gay Male Experience. Measurement and Evaluation in Counseling and Development. 2000;33(2):66–90.

2. Mohr JJ, Kendra MS. Revision and extension of a multidimensional measure of sexual minority identity: the Lesbian, Gay, and Bisexual Identity Scale. J Couns Psychol. 2011;58(2):234–45.

3. Ross MW, Rosser BR. Measurement and correlates of internalized homophobia: a factor analytic study. J Clin Psychol. 1996;52(1):15–21.

4. Smolenski DJ, Diamond PM, Ross MW, Rosser BRS. Revision, criterion validity, and multigroup assessment of the reactions to homosexuality scale. J Pers Assess. 2010;92(6):568–76.

5. Herek GM, Gillis JR, Cogan JC. Internalized stigma among sexual minority adults: Insights from a social psychological perspective. J Couns Psychol. 2009;56(1):32–43.

6. Herek GM, Cogan JC, Gillis JR, Glunt EK. Correlates of internalized homophobia in a community sample of lesbians and gay men. Journal of the Gay & Lesbian Medical Assn. 1998;2:17–25.

7. Del Pino HE, Steers WN, Lee M, McCuller J, Hays RD, Harawa NT. Measuring Gender Role Conflict, Internalized Stigma, and Racial and Sexual Identity in Behaviorally Bisexual Black Men. Arch Sex Behav. 2022;51(2):1019–30.

8. Nungesser LG. Homosexual acts, actors, and identities. New York, NY: Praeger; 1983.

9. Shidlo A. Internalized Homophobia: Conceptual and Empirical Issues in Measurement. In: Greene B, Herek G, editors. Lesbian and Gay Psychology: Theory, Research, and Clinical Applications. 2455 Teller Road, Thousand Oaks California 91320 United States: SAGE Publications, Inc; 1994. p. 176–205.

10. Neilands TB, Steward WT, Choi KH. Assessment of stigma towards homosexuality in China: a study of men who have sex with men. Arch Sex Behav. 2008;37(5):838–44.

11. Korhonen CJ, Flaherty BP, Wahome E, Macharia P, Musyoki H, Battacharjee P, et al. Validity and reliability of the Neilands sexual stigma scale among Kenyan gay, bisexual, and other men who have sex with men. BMC Public Health. 2022;22(1):754.

12. Díaz RM, Ayala G, Bein E, Henne J, Marin B V. The impact of homophobia, poverty, and racism on the mental health of gay and bisexual Latino men: findings from 3 US cities. Am J Public Health. 2001;91(6):927–32.

1. The original Internalized Homophobia Scale (IHP) was according to Herek et al. (5) originally derived from the Diagnostic and Statistical Manual of Mental Disorders-3rd edition by John Martin. The original scale was not identified during the systematic search. [↑](#footnote-ref-1)
2. Díaz et al. (12) introduced the original scale in a sample of gay and bisexual Latino men, but did not report psychometric properties. The instrument therefore did not meet our inclusion criteria and is not included in the main review; it is cited here to document the source for later validated adaptations. [↑](#footnote-ref-2)
