# Supplementary material for: Measurement instruments for sexual identity minority stress in adults: A scoping review
Source: PLoS One. 2026 Feb 12;21(2):e0342420. doi: 10.1371/journal.pone.0342420 (PMC12900343; doi:10.1371/journal.pone.0342420)
Supplement: Attachment — Submitted filename: Response to reviewers.docx [file pone.0342420.s006.docx]

**Point-by-point responses to the editors’ and reviewers’ comments**

**Editor,** Anupam Joya Sharma

**I appreciate the authors for this manuscript which attempts to address an important and timely topic, and a comprehensive review of this nature is indeed much needed. However, the current version requires substantial revisions to enhance its clarity, depth, and overall contribution to the literature.**

Thank you very much for the positive feedback. We carefully considered each comment and improved the manuscript.

**Introduction**

**The introduction should be expanded to provide a clearer framing of the study. Specifically, the statement “However, such a focus potentially neglects the multifaceted experiences of LGBTQ individuals, extending beyond sexual health inequalities” needs elaboration. The authors should clarify what is meant by “neglect” and specify the kinds of inequalities or experiences that have been overlooked in prior work. This will help set a stronger rationale for the review.**

Thank you for your suggestion. We expanded the introduction to clarify what we mean by “neglect” and to specify overlooked domains, while keeping the section concise. The revised passage now reads (l. 45-53):

*The pursuit of equal rights by Lesbian, Gay, Bisexual, and Queer (LGBQ) activists has raised awareness of health disparities within this community, initially emphasized on the sexual health of gay men due to the HIV/AIDS epidemic (1). However, such a focus potentially neglects the multifaceted experiences of LGBQ individuals, extending beyond sexual health inequalities. LGBQ individuals face unique challenges such as structural stigma (2, 3), barriers to care (4, 5), and discrimination and violence because of their sexual orientation (6). Current research increasingly reveals these challenges and distinct health concerns within this group, including higher rates of depression, anxiety, substance abuse disorders, and suicidal tendencies compared to their heterosexual counterparts (7–10).*

**Meyer’s minority stress model is mentioned very superficially but not well discussed. The literature review should engage more deeply with this model, explaining how minority stress has been conceptualized in earlier studies and synthesizing the evidence around it. This should then be linked explicitly to the gap that this review is aiming to address.**

Thank you for this helpful guidance. We substantially revised the introduction to provide a structured account of Meyer’s model, detailing distal, proximal and resilience processes and their mechanisms, and summarizing empirical support. We also added a concise synthesis of influential extensions and related frameworks to clarify mechanisms and link this overview to the measurement gap that the scoping review addresses. The minority stress model figure is moved to the introduction as Figure 1 to support reader orientation; the PRISMA flow remains in the methods section (p. 3ff).

**Methods**

**The methods section requires more detail with better transparency and reproducibility. For example, the authors should describe all components of the process, including what the data extraction sheet looked like (attachment is needed), how data extraction was performed, whether a risk of bias assessment was conducted (if not why), and how decisions were made during review. These are very important elements that currently appear to be missing.**

Thank you for this suggestion. We've updated the methods section to better explain our selection process and provide the data extraction sheets as Supplement 3. Also, we've integrated a statement on positionality at the end of the review (l. 711-720).

Consistent with PRISMA-ScR guidance, our objective was to map measurement instruments rather than to appraise study quality. Therefore, a formal risk-of-bias assessment was beyond the scope of this review. We clarified this point in our Discussion (l. 557-558):

*Consistent with PRISMA-ScR guidance (60), no risk-of-bias assessment was conducted for this scoping review.*

**Results**

**The results section is underdeveloped and should provide a richer unpacking of the findings. It should discuss the constructs studied, the contexts in which they were applied, and whether their validity and reliability were assessed or established using validated scales.**

Thank you for the constructive feedback. We’ve updated the Results to elaborate more on the constructs of the measurement instruments that were previously only listed as bullet points (p. 15f).

Thank you for the suggestion on contextualization. We now report, for each psychometric study, the place and setting of data collection in Table 1 (previously omitted for brevity) to make the development/validation context explicit. Because psychometric studies rarely specify the intended context of use for the measurement instruments, we document the empirical settings as the best available proxy. Most instruments were developed or validated in the United States and Europe (predominantly high-income settings), and we note this concentration and its implications in the Discussion (l. 564-565):

*Most instruments were developed or validated in the United States and Europe, that is, predominantly high-income settings.*

Thank you also for your comment on validity and reliability. We agree that a more detailed discussion of validity and reliability would be informative to our readers. However, as a scoping review whose aim is to provide an overview of all available instruments, it is not an objective of this work to include only studies with established psychometrics or to synthesize measurement properties. Furthermore, the reporting and assessment of validity and reliability in the included studies is highly heterogeneous and most informative when accompanied by a formal assessment of the risk of bias in the underlying evidence - an undertaking that requires a dedicated systematic review. We have highlighted the need for such a systematic review of measurement properties (including assessment of bias potential) as a clear priority for future work (please see below).

**If adaptations of constructs were made in the included studies, the authors should describe these adaptations and discuss any evidence of limitations or challenges in their use.**

Thank you for your comment. We’ve updated this section, also in response to the suggestions of reviewer 1, and included a table placing original instruments alongside their adaptations in Supplement 4. The revised text in the manuscript now reads (l. 500-502):

*Across adapted measures, the most common modifications were item reductions and revisions of measurement constructs. A side-by-side comparison of these adaptations is presented in Supplement 4.*

Consistent with the remit of a scoping review, we did not undertake instrument-by-instrument evaluations of the psychometric properties for adapted (or original) measures; this is outside the scope of this review and would require a systematic evaluation, as is characteristic of systematic reviews. We indicated this as a priority for future work (see the comment after the next one).

**Discussion**

**Similarly, the discussion section should be substantially strengthened. It should clearly articulate what this review contributes to the field and how it advances current knowledge. At present, the discussion feels cursory and does not fully capitalize on the findings. Lessons learned would be better integrated into the results or discussion sections, rather than being presented in the introduction.**

Thank you for these suggestions. We’ve substantially rewritten the discussion to meet these points. “Lessons learned” are now integrated in the discussion (l. 552-564) and we’ve included a new section “Implications for practice” to better illustrate the practical relevance of our findings. (p. 26f). In brief, the new section explains that our scoping review helps clinicians and researchers select measures aligned with their target construct, population, setting, and language. It underscores the need for clearer, transparent construct definitions as measurement in LGBQ health increasingly draws on frameworks beyond minority stress theory (e.g., microaggressions, social capital). To reduce jingle-jangle fallacies and improve comparability, we encourage explicit construct specification and propose using decentralized construct taxonomy (DCT) specifications, proposed by Peters and Crutzen (54) to map instruments to constructs, link conceptually similar tools, and clarify non-equivalences, thereby advancing harmonization of measurement in sexual minority stress research.

**Finally, the paper would benefit from a thorough reflection on the validity and reliability of the constructs examined across the reviewed studies, as this is central to the credibility of the conclusions drawn.**

Thank you for this important point. We agree that a comprehensive appraisal of the validity and reliability of measurement instruments is critical. However, such an assessment lies beyond the scope and intent of this scoping review. Given the methodological heterogeneity of psychometric investigations and the number of instruments, the appropriate next step is a dedicated systematic review with formal appraisal of evidence, including risk-of-bias assessment. We now state this explicitly in the Discussion as a priority for future work (l. 683-685).

*A dedicated systematic review is therefore warranted to conduct a formal psychometric evaluation, including risk-of-bias assessment and grading of certainty of evidence, to identify instruments best suited for research and practice.*

**While the topic is important and this review has the potential to be a valuable contribution, substantial revisions are needed to deepen the analysis, clarify the methodology, and strengthen the interpretation of results.**

Thank you for the constructive guidance and the opportunity to revise. We have substantially updated the manuscript to deepen the analysis, clarify the methodology, and strengthen the interpretation of results, and we hope the revisions align with PLOS ONE’s expectations.

**Reviewer 1**

**The purpose of this paper was to systematically map measurement instruments for sexual minority stress among LGBQ populations. The focus of the study fits within the scope of the journal and is of relevance to its readership. Below, I note specific areas to strengthen the manuscript and note concerns regarding implications of the study findings. I wish the authors the best of luck as they continue to move this research forward.**

Thank you very much for the positive feedback.

**Introduction**

1. **Overall, the Introduction is brief and cursory. It would benefit from additional details about the different constructs within the Minority Stress model and how those constructs may be measured to further elaborate the need for the scoping review. This is particularly important given that the Results are organized by constructs.**

Thank you for your comment. We improved the introduction and expanded the elaboration of the factors included in the Minority Stress Model. To better explain the central constructs, we now clearly define distal stressors, proximal stressors, and resources within the model. We have marked the terms in italics that are central to the results section to provide our readers with more guidance (p. 3ff).

2. **It is unclear why the “Lessons Learned” section is in the Introduction as this is better suited for the Discussion/Conclusion.**

Thank you for this helpful suggestion. We agree that a “Lessons learned” section is more appropriate for the Discussion. Accordingly, we removed it from the Introduction and integrated the content into the Discussion (l. 552-564). The revised passage now reads:

*Our scoping review has identified an abundance of measurement instruments, surpassing initial expectations of a limited number of measurement instruments in the field of minority stress. This array of instruments highlights the complexity and depth of research in the field of sexual minority stress. However, our review cannot determine which measurement instrument is most suitable for addressing research questions related to minority identity in terms of psychometric properties. Consistent with PRISMA-ScR guidance (60), no risk-of-bias assessment was conducted for this scoping review.*

*We observed that there are numerous measurement instruments available for internalized homonegativity and stigmatization. However, aligning the name and construct of the measurement instruments proved challenging with the risk of falling into the jingle-jangle fallacy. The importance of meticulous examination and comparison of measurement tools became evident. Further, our review revealed a predominant research focus on men in the existing literature. In contrast, instruments targeting women and bisexual individuals, are relatively rare.*

**Methods**

1. **More information regarding the independent reviewers for the stages of screening would be helpful – are their any parts of their background (e.g., academic, personal) that would influence screening? For example, anything regarding reflexivity/positionality**

Thank you for your feedback. We agree that more information is helpful for our readers given the topic of this review. We included a positionality statement at the end of the document to not overload the methods section (l. 711-720):

*Positionality statement*

*All independent reviewers involved in screening and data extraction are researchers trained in evidence synthesis with disciplinary backgrounds in psychology, public health, and epidemiology, working within Western European public institutions. We approach the topic as scholarly allies rather than community representatives and do not claim lived-experience authority. We recognize that our personal background as well as our disciplinary training, institutional context, and the English/German scholarly lens can shape interpretation. We therefore employed rigorous methodology and reflected on potential normative assumptions and made the fewest possible adjustments to the included studies’ descriptions and documented any necessary clarifications transparently (see Data Analysis and table footnotes).*

2. **Details regarding the consensus process would also be helpful. Who resolved conflicts?**

Thank you for your comment. We now detail this process further (l. 217-220):

*At the second stage, conflicts on the in- and exclusion of references were resolved in consensus by thorough discussion between the involved screeners and, if no consensus could be reached, through discussion with all screeners (MMK, SC, SF, AJ, MSix), with resolution by majority vote.*

3. **Can the authors please provide the data extraction form?**

Thank you for this suggestion. We now provide the data extraction form in Supplement 3.

**Results**

1. **There are a series of measures (page 10-11, lines 214-238) that are listed at bullet points. It may be more appropriate to describe these in a narrative format and provide additional details regarding the constructs they address.**

Thank you for your suggestion. We reworked that passage and now provide more information on the included constructs (p. 15f).

2. **Reference(s) are needed to support the claim made on lines 240-243.**

Thank you for mentioning the missing references. We’ve added the relevant reference to Meyer’s minority stress model which were missing before (l. 394-398):

*The following instruments capture constructs that do not directly map onto Meyer’s minority stress model (11, 12, 41) but were included due to their conceptual relevance to minority stress experiences. While they do not assess distal or proximal stressors as defined by Meyer (11), they address themes such as intraminority dynamics, social support, and identity-related cognitions, which may influence or co-occur with minority stress.*

3. **For the measures listed as having been adapted (page 14, line 328), it would be helpful to know what the scope of the adaptation(s) were.**

Thank you for your comment. We have updated this section and included a table putting original measurement instruments next to their adaptations in Supplement 4. The revised section in the manuscripts now reads (l. 500-502):

*Across adapted measures, the most common modifications were item reductions and revisions of measurement constructs. A side-by-side comparison of these adaptations are presented in Supplement 4.*

**Discussion**

1. **Line 384: study is described as a systematic literature review, but was initially framed as a scoping review.**

Thank you for noting this inconsistency. We corrected the phrasing in that paragraph and ensured consistent use of “scoping review” throughout the manuscript. The revised sentence (l. 552-553) reads:

*Our scoping review has identified an abundance of measurement instruments, surpassing initial expectations of a limited number of measurement instruments in the field of minority stress.*

2. **The authors state that there were limited instruments for assessing positive aspects of minority identity, but it does not appear as though these types of measures were explicitly sought out for based on the search strategy.**

Thank you for your comment. Our search strategy combined broad population and measurement terms with more specific minority-stress-related terms (discrimination, stigmatization, homophobia, prejudice). We acknowledge that, within the more specific component, we did not prespecify positive-identity keywords (e.g., pride, community connectedness, identity affirmation, resilience). We expected such instruments to be captured by the generic arm of the strategy under the minority stress model umbrella but agree that this omission could contribute to the low yield of positive-identity measures. Accordingly, we have deleted this claim in the beginning of the Discussion and revised the section on this point (please see below for the revisions on this section)

3. **It is not until the Discussion that we know that Dissertations were included. Typically, peer-reviewed sources are only included. Rationale for including Dissertations is necessary as this can limit the rigor/validity of the review.**

Thank you for mentioning this point. A hand search for grey literature was performed through websites. We’ve clarified this part in the Methods section which now reads (l. 207-211):

*The search was conducted across Medline, Embase, PsycInfo, PSYNDEXplus, PTSDpub and Web of Science. Additionally, a hand search for grey literature in the GESIS Open Access Repository for Measurement Instruments and the GLBTQ+ Alliance in Social and Personality (GASP) Measures Database was performed and the reference lists of included articles were examined for further relevant publications.*

We’ve also clarified the inclusion of studies independent of source under ‘Study selection’ (l. 232-233):

*No restrictions were applied with regard to the year of publication or the publication medium.*

However, most dissertations and grey literature were retrieved via PsycInfo and, upon meeting our a priori inclusion criteria (psychometric study reporting on the development or evaluation of measurement properties), were included to avoid omitting original instrument reports and to reduce the risk of publication bias. We agree that grey literature varies in quality, but we would argue for the scientific legitimacy of dissertations in particular. Theses are evaluated through rigorous review processes, with evidence demonstrating their high quality^[1]^.

To increase transparency, we revised the Results section and now report a breakdown: 16 instruments were documented in grey literature. Of these, seven were available only in theses (e.g., EAIMS, PANQ, IHRM, ISPS); four also had psychometric reports in journals (e.g., LGBT Minority Stress Measure, NOS, MAGI-MSV, BMMS-W); two were identified only in conference proceedings with detailed psychometrics (GALOSI-E, GALOSI-F); and book or book-chapter sources were identified for NHAI, NHAI-SR, and SLIHS (alongside dissertation material). We have added this information to the manuscript and clearly flag instruments supported exclusively by grey literature (l. 270-290):

*Most psychometric studies were reported in peer-reviewed journals. However, 16 instruments were documented in grey literature. Seven were available only in theses:*

*1.	Emerging Adult Inventory of Minority Stress (EAIMS) (42)*

*2.*	*The Positive Aspects of Nonheterosexuality Questionnaire (PANQ) (123)*

*3.	The Internalized Heterosexist Racism Measure (IHRM) (179)*

*4.	The Gay Identity-based Shame and Pride Scales (148)*

*5.	Internalized Sexual Prejudice Scale (ISPS) (66)*

*6.	Chinese and Chinese American Sexual Minority Women Internalized Heterosexism (CCSMW-IH) Scale (129)*

*7.	Bisexual Microaggressions Scale (96).*

*Four measurement instruments also had psychometric reports in journals:*

*1.	LGBT Minority Stress Measure (44, 45)*

*2.	Nebraska Outness Scale (NOS) (56, 115)*

*3.	Multi-Axial Gay Men’s Inventory (Men’s Short Version, MAGI-MSV) (64, 65)*

*4.	Bisexual Microaggression and Microaffirmation Scales for Women (BMMS-W) (97–99).*

*The Nungesser Homosexual Attitudes Inventory (NHAI) (23) and the Shidlo-revised NHAI (NHAI-SR) (29) were originally published as a book and later further validated in a dissertation (66). On the other hand, the Self-Identified Lesbian Internalized Homophobia Scale (SLIHS) was originally published as a dissertation and later also published in a book (67, 68). Finally, two instruments, GALOSI-E and GALOSI-F (69), were identified only in conference proceedings that reported detailed psychometric results.*

4. **For the section beginning on line 411, I again raise the concern of positive aspects of minority identity not being included in the search, thus statements about the limited number of these types of measures should be tempered.**

Thank you for your comment. As stated above, we agree that the omission of positive-identity keywords from our search strategy could contribute to the low yield of relating measures. For this section, we have moderated and contextualized more the claim to avoid over-interpreting the apparent scarcity (l. 586-599):

*Contrastingly, positive facets of minority identity, specifically self-acceptance and pride, are less represented in the literature but acknowledged as areas requiring further investigation (204, 81, 205). This pattern should be interpreted with caution: although our search combined broad population and instrument terms to capture sexual-identity minority stress measures (see Supplement 2), we did not explicitly include keywords for positive identity constructs, which may have limited retrieval. Nonetheless, within the included set, our temporal mapping (Fig 4) shows that earlier instruments targeted negative constructs (e.g., internalized homonegativity, stigmatization, identity concealment), with positive constructs (e.g., self-acceptance and pride) emerging later. Furthermore, this observation aligns with Meyer’s call for stronger integration of resilience within health research on LGBT populations (41). The minority strength framework proposed by Perrin et al. (204) suggests that factors such as identity pride and community connectivity can mediate favorable health outcomes in LGBTQ populations. Therefore, a comprehensive understanding of the LGBQ experience thus necessitates the inclusion of both stress and resource-based perspectives (11, 41, 206).*

5. **No implications for practice are presented. It is unclear what the findings of the scoping review contribute to the field and/or advance measurement of minority stress.**

Thank you for your comment. We’ve updated the discussion and now include a section “Implications for practice” in our discussion (p. 26f). In brief, the new section explains that our scoping review helps clinicians and researchers select measures aligned with their target construct, population, setting, and language. It underscores the need for clearer, transparent construct definitions as measurement in LGBQ health increasingly draws on frameworks beyond minority stress theory (e.g., microaggressions, social capital). To reduce jingle-jangle fallacies and improve comparability, we encourage explicit construct specification and propose using decentralized construct taxonomy (DCT) specifications, proposed by Peters and Crutzen (54) to map instruments to constructs, link conceptually similar tools, and clarify non-equivalences, thereby advancing harmonization of measurement in sexual minority stress research.

**Reviewer 2**

**Abstract:**

1. **Needs further enrichment E.g. stigma is missed, T is missed.**

Thank you for this comment. We’ve updated the abstract to include explicitly stigma as relevant construct (l. 28-31):

*Selection criteria focused on instruments measuring minority stress in form of stigmatization, discrimination, victimization, internalized homophobia, expectations of rejection, identity concealment, as well as positive aspects of minority identity among adult LGBQ populations.*

Regarding the inclusion of T: Our review focuses on sexual identity minority stress; accordingly, we use LGBQ to denote the target population, independent of gender identity. We also added in the abstract, that gender-identity minority stress lies beyond the scope of this review (l. 31-32):

*Gender-identity-related stressors were outside the scope.*

**Introduction:**

1. **Is weak for its contents E.g What are the factors associated yet and initiaties for preention.**

Thank you for this comment. We strengthened the introduction by detailing distal, proximal and resilience factors within the model. We also summarized adapted frameworks to the minority stress model that inform more clearly prevention and intervention targets, including cognitive, affective, and interpersonal mediators. This clarifies where interventions may act while keeping the focus on measurement (p. 3ff).

2. **E.g2. line 53-54 lacks references**

Thank you for bringing this to our attention. In revising the introduction, we added a new heading ‘Proximal stress factors’ and included references surrounding our statements (p. 4ff). The sentence in question was specifically completed with a reference to Meyer’s minority stress model (l. 76-77):

*Proximal processes can exacerbate these stressors, such as the anticipation of negative events and the internalization of LGBQ-hostile ideologies (11).*

**Methods**

1. **Sexual identity minority stres should be operationalzed.**

Thank you for highlighting the need to operationalize sexual identity minority stress. We now define Meyer’s minority stress model explicitly though stressors and processes specific to sexual-identity minority status, comprising (i) distal stressors, e. g., experiences of victimization, and discrimination; (ii) proximal processes, e. g., internalized homonegativity, expectations of rejection/vigilance, and concealment; and (iii) resilience, e. g., pride and community connectedness (Introduction, p. 3ff).

2. **Inclusion criteria is not complete E.g. Language, and year of publication.**

Thank you for the comment. The language criterion was already specified (l. 232). We have now added the missing time-frame statement in the Methods section (l. 232-233):

*Only articles in English and German were considered. No restrictions were applied with regard to the year of publication or publication medium.*

3. **What does 'validated studies' mean? How was the process? What is the justification behind it?**

Thank you for flagging this. We identified a terminology inconsistency: the term “validation studies” appeared in the Data Analysis section, whereas elsewhere we used “psychometric studies”. We have now standardized the wording to “psychometric studies”, e.g., studies reporting on the development or validation of a measurement instrument, throughout the manuscript.

4. **What does language validation Vs inclusion of other languages like Polish and Italian means?**

Thank you for this question. In this review, “language validation” refers to the translation and cross-cultural adaptation of an existing instrument into a target language followed by psychometric testing. Such versions (e.g., a Portuguese translation of an English instrument) were attributed to the parent measure and not counted as separate instruments to avoid double counting. By contrast, instruments originally developed in languages other than English or German (e.g., Polish or Italian) were counted as distinct instruments. Study eligibility was determined by the publication language (English or German), irrespective of the instrument’s language. The Methods section has been updated to clarify this distinction (l. 258-260):

*Language validations of measurement instruments, defined as translation and cross-cultural adaptation of an existing instrument followed by psychometric testing, were not treated as separate entities in this analysis to prevent an overrepresentation of the same instrument.*

**Results**

1. **Lacks time sequence or evolution**

Thank you for this helpful suggestion. We have updated the section on adaptations of minority stress measurement instruments and included a table putting original measurement instruments next to their adaptations in Supplement 4. The revised section in the manuscripts now reads (l. 500-502):

*Across adapted measures, the most common modifications were item reductions and revisions of measurement constructs. A side-by-side comparison of these adaptations is presented in Supplement 4.*

We also provide now a temporal analysis of instrument development. Each instrument is coded by the year of its first publication and displayed in a construct-by-year heat map (Fig. 4). The Methods describe the coding and visualization (l. 249–250). In the Results, we summarize the evolution: early work (1980s–1990s) concentrated on internalized homonegativity and concealment; from the early 2000s development diversified (e.g., stigmatization, discrimination); after 2010 growth accelerated, with stigmatization becoming the most frequently operationalized domain (l. 513–520).

*When mapped against the year of the first publication of a measurement instrument, Fig 5 illustrates how the development of instruments has shifted across minority stress-related constructs over time. Early measures, appearing in the 1980s and 1990s, predominantly targeted internalized homonegativity and identity concealment, reflecting the initial focus on intrapersonal processes. From the early 2000s onwards, instrument development diversified to include stigmatization, discrimination, and other constructs elaborated above. This diversification accelerated markedly after 2010, with the 2010s and 2020s showing the greatest density of new measures. During this period, stigmatization emerged as the most frequently operationalized construct.*

*Fig 5. Heat map of measurement instruments by construct and year*

2. **Contextualization E.g. Countries of.lowincome, middle income and high income.**

Thank you for this suggestion. We now report, for each psychometric study, the place and setting of data collection in Table 1 (previously omitted for brevity) to make the development/validation context explicit. Because psychometric studies rarely specify the intended context of use for the measurement instruments, we document the empirical settings as the best available proxy. Most instruments were developed or validated in the United States and Europe (predominantly high-income settings), and we note this concentration in the Discussion (l. 564-565):

*Most instruments were developed or validated in the United States and Europe, that is, predominantly high-income settings.*

3. **Inconsistency E.g. LGBQ Vs LGBTQ, absence of stigma in some sections**

Thank you for this helpful comment. Our review focuses on sexual identity minority stress; accordingly, we use LGBQ to denote the target population, independent of gender identity. We have conducted a global consistency pass to ensure LGBQ is used throughout when referring to our scope, and we retain LGBTQ/LGBTQI+ only where this terminology is used in the original sources or instrument names. We now state this explicitly in the Terminology/Scope note and in the Positionality Statement (l. 711-720):

*All independent reviewers involved in screening and data extraction are researchers trained in evidence synthesis with disciplinary backgrounds in psychology, public health, and epidemiology, working within Western European public institutions. We approach the topic as scholarly allies rather than community representatives and do not claim lived-experience authority. We recognize that our personal background as well as our disciplinary training, institutional context, and the English/German scholarly lens can shape interpretation. We therefore reflected on potential normative assumptions and made the fewest possible adjustments to the studies’ descriptions included and documented any necessary clarifications transparently (see Data Analysis and table footnotes).*

In line with Meyer’s framework, we use “stigma” only where it is the relevant construct (e.g., enacted/structural stigma), and we label related but distinct constructs precisely (e.g., internalized homonegativity, concealment, expectations of rejection). To make this taxonomy transparent, we expanded the Introduction to delineate distal vs proximal processes and indicate explicitly where “stigma” applies (see Introduction, p. 3ff). We hope these clarifications address the concern without overgeneralizing the term.

**Discussion**

1. **Discussion need more references and explanation.**

Thank you for this helpful comment. We have significantly expanded the discussion, added several citations, and clarified our argumentation throughout. Specifically, we have strengthened the points on the theoretical foundation of instruments and linked them to work on decentralized construct taxonomies; and we have anchored our review more firmly in the relevant literature (e.g., microaggressions; social capital). This is primarily reflected in a new section titled “Implications for Practice” (p. 26f), which is supported by references and summarizes specific conclusions for instrument selection and construct specification.

2. **THE reference cited should be references**

Thank you for your comment. We updated the headline of the reference section to “References” (l. 735).

1. McLeod BD, Weisz JR. Using dissertations to examine potential bias in child and adolescent clinical trials. J Consult Clin Psychol. 2004 Apr;72(2):235-51. doi: 10.1037/0022-006X.72.2.235.
